# Epigenetic regulation of regulatory T cells in patients with abdominal aortic aneurysm

**DOI:** 10.1002/2211-5463.12643

**Published:** 2019-05-14

**Authors:** Qian Xia, Jian Zhang, Yanshuo Han, Xiaoyu Zhang, Han Jiang, Yu Lun, Xiaoyu Wu, Qingwei Gang, Zhimin Liu, Dittmar Böckler, Zhiquan Duan, Shijie Xin

**Affiliations:** ^1^ Department of Vascular Surgery The First Hospital China Medical University Shenyang China; ^2^ Department of Vascular and Endovascular Surgery University of Heidelberg Heidelberg Germany

**Keywords:** abdominal aortic aneurysm, DNA methylation, epigenetics, histone modifications, regulatory T cells

## Abstract

Abdominal arterial aneurysm (AAA) shares many features with autoimmune diseases and appears to be a T‐cell‐mediated process. In addition, certain epigenetic changes, including DNA methylation, are associated with AAA. In this study, we investigated epigenetic modifications in regulatory T cells (Tregs) from AAA patients. We used flow cytometry to sort FOXP3^+^ CD4^+^ CD25^+^ Tregs from the peripheral blood of AAA patients and from healthy controls (HC), and then detected DNA methylation and histone modifications by ELISA. The DNA methylation rate of Tregs was significantly higher in AAA patients than in the HC group (0.159 ± 0.08% *vs* 0.098 ± 0.03%, *P* < 0.05), while the acetylation rates of H3 and H3K9 histones were lower in the AAA than in the HC group. We also examined the expression of mRNA encoding enzymes that catalyze making and removing epigenetic modifications by real‐time PCR: we found that mRNA levels of DNA methyltransferase (DNMT) 1 and DNMT3A were higher in the AAA than in the HC group, mRNA levels of methyl‐CpG‐binding domain protein (MBD) 2 and MBD4 were higher in the AAA than in the HC group (MBD2: 6.21 ± 2.57 *vs* 3.04 ± 1.45; MBD4: 7.76 ± 3.48 *vs* 4.97 ± 3.10; both *P* < 0.05), and mRNA levels of histone deacetylase (HDAC) 1 and HDAC5 were significantly up‐regulated in the AAA compared with the HC group (HDAC1: 2.17 ± 1.18 *vs* 1.51 ± 0.99; HDAC5: 1.35 ± 0.49 *vs* 0.94 ± 0.76; both *P* < 0.05). Together, our results reveal that rates of DNA methylation and histone modifications of Tregs are significantly altered in AAA patients.

**Abbreviations**
AAA: abdominal aortic aneurysm
DNMT: DNA methyltransferase
FOXP3: forkhead box P3
HAT: histone acetyltransferase
HC: healthy control
HDAC: histone deacetylase
HMT: histone methyltransferases
MBD: methyl‐CpG‐binding domain protein
PBMC: peripheral blood mononuclear cell
SD: standard deviation
Treg: regulatory T cell

Aneurysms are arterial lesions that cause a persistent extension of an artery segment; they are more common in older men. They are commonly seen in the abdominal aorta (abdominal aortic aneurysm; AAA) and the lower extremity. Such arterial lesions can be caused by multiple factors including auto‐immune disease, inflammation, arterial trauma, lipid deposits, hypertension and even smoking. Rupture of an AAA is highly dangerous 1. The incidence and the prevalence of AAA in China have been increased during the past decade 2.

AAA shares many features with autoimmune diseases, including genetic predisposition, organ specificity and chronic inflammation 3. Increasing evidence has shown that an immune response plays an important role in aneurysmal disease development, and AAA appears to be a T‐cell‐mediated process 4, 5. Vascular and molecular biology studies have revealed the role of the adaptive and innate immune systems in the initiation and propagation of the inflammatory response in aortic or aneurysmal tissue 6. Our previous studies have revealed impaired Fas‐induced apoptosis of T lymphocytes and a functional deficiency of regulatory T cells (Tregs) in AAA patients, which supports the abnormal modification of peripheral T cells in AAA tissue and implies an important role of autoimmunity in the etiology of AAA 7, 8. However, the mechanisms modulated by Tregs in AAA were still unknown.

Epigenetics is defined as the research on genome properties that are not explained by the primary DNA sequence, but are caused by modifications of the DNA and/or its associated proteins 9. Epigenetic modifications include DNA methylation, histone modifications and the role of non‐coding RNA, which can directly interact with the primary nucleotide sequence and regulate gene expression. Changes of DNA methylation have been reported to be related to AAA 5, 10, 11, 12. However, very few studies have investigated the functions of histone modifying enzymes [histone deacetylases (HDACs), histone methyltransferases (HMTs) and histone acetyltransferases (HATs)], which have been shown to be related to AAA pathology in humans 13. Thus, the epigenetic changes in the pathogenesis of AAA have been lacking. This study was designed to investigate whether DNA methylation and histone modifications were altered in Tregs of AAA patients.

## Materials and methods

### Patients

Thirty‐two AAA patients (the AAA group, 27 men and five women, mean age: 63.9 ± 8.1 years) at Department of Vascular Surgery, The First Hospital, China Medical University (from September 2010 to July 2013) were prospectively recruited into the study. All patients and control subjects gave written informed consent. AAA with a diameter > 50 mm was included, as measured by a computed tomography scan or magnetic resonance imaging. Patients with the following complications were excluded: atherosclerotic changes, Ehlers–Danlos syndrome, Marfan syndrome, other known vascular disorders, connective tissue disorders and medical history of other autoimmune diseases. Thirty age‐ and sex‐matched healthy controls (HCs) who received annual regular physical examinations were included as the HC group (26 men and four women, mean age: 60.5 ± 11.2 years). All the controls had a healthy peripheral vascular system, without medical history of aneurysm, type A aortic dissection, or other vascular disorders. In addition, cancer, infection, and any other immune‐mediated diseases were also excluded. The study conformed to the guidelines set by the Declaration of Helsinki. All experimental protocols were approved by the Institutional Review Board at China Medical University (Shenyang, China).

### Isolation of peripheral blood mononuclear cells and Tregs

Peripheral blood was collected in 40 mL anticoagulant tubes. Thereafter, peripheral blood mononuclear cells (PBMCs) were isolated with density‐gradient centrifugation in Ficoll–sodium diatrizoate (Ficoll‐Hypaque, Haoyang TBD, Co., Tianjin, China). The cells were washed with PBS and CD4^+^ and CD25^+^ Tregs were sorted by flow cytometry. The extract of PBMCs and residual liquid Sortin were blended, and then 50 μL FITC–CD4 and 50 μL APC–CD25 fluorochrome were added into the mixture for 20 min in the dark. The dyed PBMCs were washed with 2 mL PBS once, and the supernatant was discarded. The cell pellet was diluted in 10 times, and kept at room temperature for 10 min in dark. Then, the liquid was centrifuged for 5 min, the supernatant was discarded, and washed twice with PBS. Finally, 50 μL forkhead box P3 (FOXP3) antibody was added into the cell pellet followed by culture for 30 min at dark, and then, CD4^+^ CD25^+^ Tregs were sorted by flow cytometry.

### DNA methylation determination

A blood genomic DNA extraction kit (Tiangen Biochemical Technology Co., Ltd, Beijing, China) was used to extract DNA, and a centrifuge column was used to separate DNA. DNA methylation rate was detected using a DNA methylation kit (Epigentek, Group. Inc., New York, NY, USA) according to the manufacturer's instructions. 5‐Methylcytosine (5‐mc) was quantitatively analyzed using an ELISA kit. Standard absorbance was measured by a microplate reader according to the formula: 5‐mc (ng) = (sample *A* − negative control *A*)/slope × 2. The 5‐mc % = 5‐mc (ng)/amount of DNA × 100% was calculated according to manufacturer's instructions.

### Histone extraction and protein quantification

Histone was extracted with a total histone protein extraction kit (Epigentek Group, Inc.) according to the manufacturer's instruction. Briefly, the cells were centrifuged at 168 ***g***, 4 °C for 5 min and suspended in diluted pre‐lysis buffer at 10^7^/mL. After centrifuging at 1509 ***g*** 4 °C for 5 min, the supernatant was discarded; the cells were re‐suspended in three volumes (about 10^7^ cells/200 μL) of cell lysate and incubated on ice for 30 min. After centrifuging at 24 149 ***g*** at 4 °C for 5 min, the supernatant was moved to a new tube; 0.3 volumes of DTT and balanced salt solution at a ratio of 1 : 500 (1 μL DTT + 500 μL balanced salt solution) was added to the supernatant. Protein concentrations were determined using the enhanced BCA Protein Assay Kit (Beyotime Institute of Biotechnology, Nanjing, China). After extraction, histones were stored at −80 °C for use.

### Determination of acetylation rate of histone H3, H3K9, H3K14 and H4

The acetylation rates of total histones H3, H3K9, H3K14 and H4 were detected by ELISA according to the manufacturer's instructions (Epigentek, Group. Inc.). The protein concentrations were adjusted to 400 ng·μL^−1^ and absorbance was measured at 450 nm wavelength using a microplate reader. The degree of histone acetylation rate was calculated according to the following formula: Acetylation % = *A*_sample well−blank well)_/*A*_control well−blank well_ × 100%.

### Real time‐polymerase chain reaction

Total RNA was extracted using an RNAiso Plus Kit (TaKaRa Biotechnology Co., Dalian, China.), and the amount and purity of the RNA were determined with spectrophotometry. High‐quality RNA samples were obtained with an *A*_260_/*A*_280_ ratio > 1.8. Total RNA was reverse‐transcribed with reverse transcriptase (PrimeScript RT Reagent Kit, TaKaRa) and oligo‐dT primer according to the manufacturer's instructions. The reaction conditions were: 95 °C for 30 s, then 95 °C for 5 s, and 60 °C for 30 s for 40 cycles. The expression of target genes was normalized to reference as previously described 14. The primers designed for real‐time PCR are listed in Table 1.

**Table 1:** The primers for real‐time PCR.

Gene	Primer	Sequence(5′–3′)
*DNMT1*	Forward	CTTCGGCAACATAATGGACA
	Reverse	CTTTGGCCAACATACAAAGCTTGA
*DNMT3A*	Forward	CCGGAACATTGAGGACATCT
	Reverse	CAGCAGATGGTGCAGTAGGA
*DNMT3B*	Forward	CCCATTCGAGTCCTGTCATT
	Reverse	GGTTCCAACAGCAATGGACT
*MBD1*	Forward	CACCCTCTTCGACTTCAAACAAG
	Reverse	CAACCTGACGTTTCCGAGTCTT
*MBD2*	Forward	AACCCTGCTGTTTGGCTTAAC
	Reverse	CGTACTTGCTGTACTCGCTCTTC
*MBD3*	Forward	CCGCTCTCCTTCAGTAAATGTAAC
	Reverse	GGCTGGAGTTTGGTTTTCAGAA
*MBD4*	Forward	TGGTGGTGCATGCCTGTAAT
	Reverse	TGAGACAGGGTCTCTCTCTGTCAT
*HDAC1*	Forward	AGACAGCTGTGGCCCTGGATA
	Reverse	GGAGCTTGAAATCTGGTCCAAAGTA
*HDAC2*	Forward	AGTCAAGGAGGCGGCAAAA
	Reverse	TGCGGATTCTATGAGGCTTCA
*HDAC4*	Forward	AAGGAGTGGACATTGCT
	Reverse	GATTCAGCAGCTCCACT
*HDAC5*	Forward	TCACTGTCACCAACTCAC
	Reverse	CAGGAATAGAGGATGTGC
*HDAC7*	Forward	CTTCTCCACAAGGACAAG
	Reverse	CTCCAGGGTTCTGTAGG
*GAPDH*	Forward	CCACCCATGGCAAATTCCCATGGCA
	Reverse	TCTAGACGGCAGGTCAGGTCCACC

### Statistical analysis

Quantitative data are presented as mean ± standard deviation (SD). The data showing a normal distribution were analyzed by *t* test. Categorical variables, including gender, smoking history, hypertension history, diabetes history and coronary disease, were analyzed by the χ^2^ test. A value of *P* < 0.05 was set as the threshold for significance. The analyses were performed using spss version 16.0 software (SPSS Inc., Chicago, IL, USA).

## Results

### DNA methylation rate and acetylation rate of histone H3 and H3K9 in Tregs were changed in AAA patients

**Figure 1:**
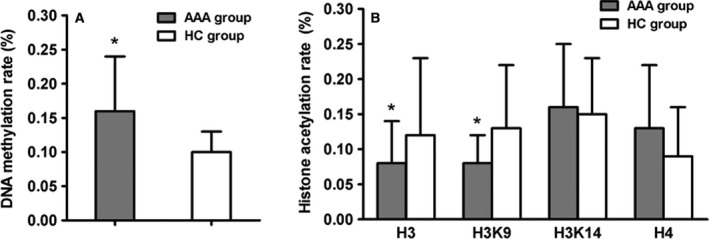
DNA methylation rate and acetylation rate of histone H3 and H3K9 in Tregs were changed in AAA patients. (A) DNA methylation rate of Tregs was up‐regulated in AAA patients; (B) Acetylation rate of histone H3 and H3K9 in Tregs was reduced in AAA patients. All data are presented as mean ± SD and analyzed by unpaired Student's *t* test. *n* = 32 in AAA group and *n* = 30 in HC group. *P < 0.05 compared with HC group.

Demographics of AAA patients and healthy controls are listed in Table 2. The sex, age, smoking history, disease history, WBC count and lymph count were comparable in the two groups. DNA methylation rate of Tregs was higher in the AAA group than in the HC group (0.159 ± 0.08% *vs* 0.098 ± 0.03%, *P *< 0.05) (Fig. 1A). The acetylation rates of histone H3 and H3K9 in peripheral blood Tregs was significantly lower in AAA patients than in HC (respectively 0.086 ± 0.06% *vs* 0.11 ± 0.099%, *P *=* *0.027 for H3; 0.08 ± 0.037% *vs* 0.125 ± 0.09%, *P *=* *0.016 for H3K9; Fig. 1B). By contrast, acetylation rates of H3K14 (AAA group: 0.163 ± 0.09% *vs* HC: 0.146 ± 0.077%, *P *=* *0.411) and of H4 (AAA group: 0.146 ± 0.03%; HC: 0.09 ± 0.012%, *P *=* *0.134) were not significantly different between the two groups.

**Table 2:** Demographics of AAA patients and healthy controls. Comparison by Unpaired Student's *t* test or χ^2^ test. WBC (white blood cell).

	AAA group (*n* = 32)	Control group (*n* = 30)	*P*
Male/female	27/5	26/4	0.303
Age (years)	63.93 ± 8.14	60.45 ± 11.17	0.916
Smoking history (n (%))	5 (27)	8 (40)	0.506
Hypertension history (*n* (%))	11 (61)	9 (45)	0.352
Diabetes history (*n* (%))	3 (16.7)	4 (20)	0.128
Coronary disease (*n* (%))	3 (16.7)	3 (15)	1
WBC count (×10^9^/L)	7.83 ± 2.56	6.59 ± 1.73	0.102
Lymph count (×10^9^/L)	1.85 ± 0.39	2.17 ± 0.62	0.11

### mRNA expression of DNA methyltransferases 1 and 3A were up‐regulated in AAA patients

**Figure 2:**
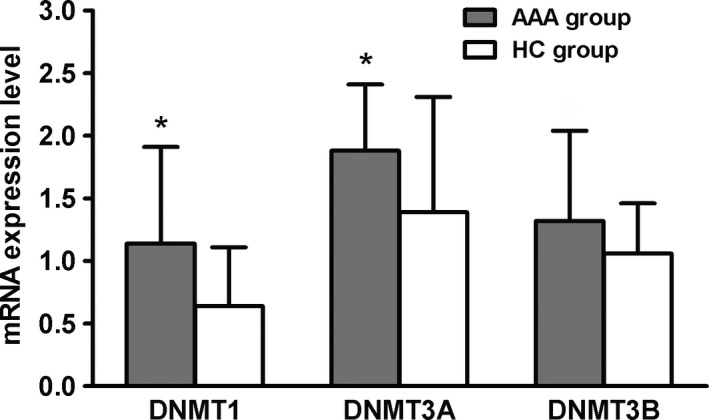
Expression of DNMT1 and DNMT3A mRNA was up‐regulated in AAA patients. All data are presented as mean ± SD and analyzed by unpaired Student's *t* test. *n* = 32 in AAA group and *n* = 30 in HC group. *P < 0.05 compared with HC group.

Three DNA methyltransferases (DNMTs) were detected using real time‐PCR. mRNA levels of DNMT1 and DNMT3A were significantly up‐regulated in the AAA group compared with the HC group (respectively 1.14 ± 0.77 *vs* 0.64 ± 0.47, *P* = 0.004 for DNMT1; and 1.88 ± 0.56 *vs* 1.01 ± 0.60, *P *< 0.05 for DNMT3A), which was consistent with the results of DNA hypermethylation. However, there was no differences regarding DNMT3B expression between two groups (AAA: 1.54 ± 1.25 *vs* HC: 1.06 ± 0.68, *P *=* *0.298; Fig. 2).

### The expression of methyl‐CpG‐binding domain proteins 2 and 4 mRNA was up‐regulated in AAA patients

**Figure 3:**
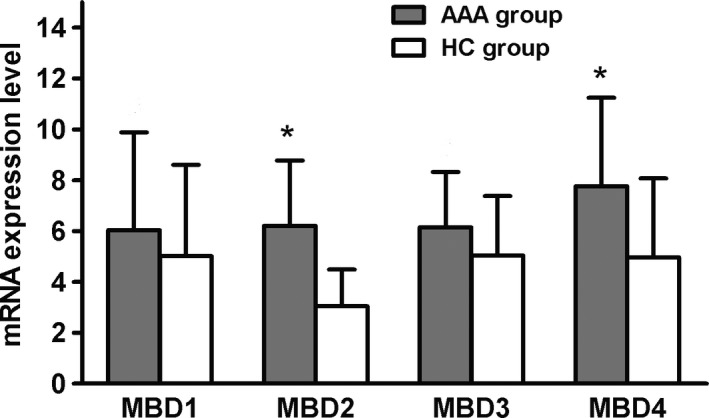
Expression of MBD2 and MBD4 mRNA was up‐regulated in AAA patients. All data are presented as mean ± SD and analyzed by unpaired Student's *t* test. *n* = 32 in AAA group and *n* = 30 in HC group. *P < 0.05 compared with HC group.

We examined mRNA expression of methyl‐CpG‐binding domain proteins (MBDs) in the two groups. Interestingly, mRNA expression in MBD2 and MBD4 was higher in the AAA group than in the HC group (respectively 6.21 ± 2.54 *vs* 3.04 ± 1.45 for MBD2; and 6.89 ± 2.24 *vs* 4.04 ± 1.75 for MBD4; both *P < *0.05). By contrast, the mRNA expression of MBD1 and MBD3 was not different between the two groups (Fig. 3).

### mRNA expression of HDAC1 and HDAC5 was increased in AAA patients

**Figure 4:**
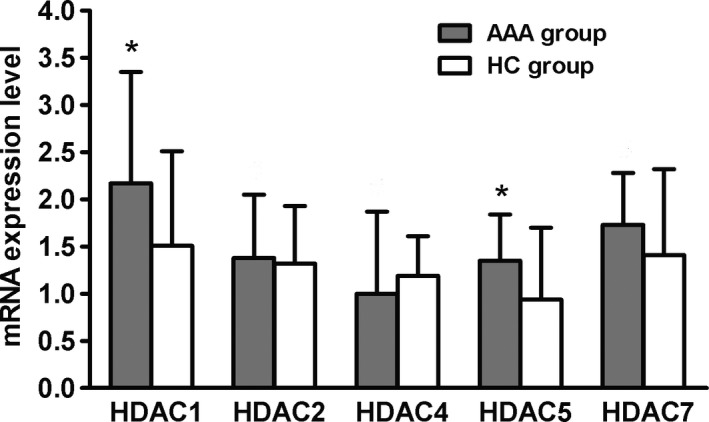
HDAC1 and HDAC5 mRNA expression was increased in AAA patients. All data are presented as mean ± SD and analyzed by unpaired Student's *t* test. *n* = 32 in AAA group and *n* = 30 in HC group. *P < 0.05 compared with HC group.

The mRNA levels in both HDAC1 and HDAC5 were higher in the AAA group than in the HC group (respectively 2.58 ± 1.66 *vs* 1.21 ± 0.9, *P *=* *0.034 for HDAC1; 1.35 ± 1.01 *vs* 0.37 ± 0.11, *P *=* *0.01, for HDAC5). The expression of HDAC2, HDAC4 and HDAC7 was not significantly different between the two groups (Fig. 4).

## Discussion

Abdominal arterial aneurysm is a complex disease that develops due to the interaction of environmental risk factors and genetic predisposition 15. DNA methylation and histone modification influence disease onset and progression as effectors of the environment, such as lifestyle or diet, and have been studied extensively in many diseases 16. In this study, we revealed that DNA methylation and histone modifications in FOXP3+ CD4+ CD25+ Tregs were different in AAA patients.

DNA methylation occurs naturally as a result of aging and cell differentiation but is also recognized as an important modifier of disease risk. DNMTs induce and maintains DNA methylation. There are two types: DNMT1 (a maintenance enzymes) and DNMT3 (a *de novo* enzyme) 17. DNMT1 recognizes hemi‐methylated sites opposite newly synthesized DNA strands in mitosis. Recognizing the role of epigenetic modifications in autoimmune diseases is crucial for a full understanding of autoimmunity. In this study, the level of DNA methylation in Tregs was significantly higher in AAA patients than in healthy subjects, indicating that Tregs were in the state of transcriptional repression, affecting the cellular function. The results were consistent with a previous study 11. On the contrary, another study pointed out that DNA methylation in peripheral T cells was significantly lower in an AAA group than a HC group 5. Thus, Toghill *et al*. 12 considered that no direct evidence yet confirms the association between DNA methylation and AAA. DNMT3 enzymes are divided into two groups: DNMT3A and DNMT3B. DNMT3A is thought to have a preference for unmethylated DNA, but DNMT3B is equally efficient at methylating unmethylated and hemimethylated CpG sites 46 17. Our results showed that DNMT3A was significantly higher in the AAA group than in the HC group, while DNMT3B was not different between the two groups, indicating that DNMT3A and 3B perform different functions in the two groups.

Previous studies identified disease‐associated changes in the expression of DNMTs and MBDs in some autoimmune diseases such as systemic lupus erythematosus and subacute cutaneous lupus erythematosus. Increased expression of DNMT1 would maintain the hypermethylation of Tregs and cause a decrease in the transcriptional activity of Tregs, resulting in insufficient numbers of cells and affecting the normal cell function. Abnormal expression of DNMT3A reveals that Treg cells in some AAA patients have aberrant methylation. The increased expression of MBD2 and MBD4 in the AAA group was also consistent with the increased rate of DNA methylation, further enhancing the main function of regulatory T cells to inhibit autoimmune diseases.

Histone modification occurs mainly during cell cycling, cell development and differentiation. The most common modification is acetylation 18. In this study, the acetylation rates of H3 and H3K9 were lower in AAA patients than in HC, which reduced the transcriptional activity and might contribute to the decrease of Tregs. These processes involve selected lysine residues in the tails of nucleosomal histones, which are induced by HAT and HDAC 9. Histone modifying enzymes, including HAT, HMT and HDACs, have been implicated in cardiovascular disease, cancer and Alzheimer's disease 19, 20. The latest study has reported that HDAC can regulate non‐histone acetylation 21; 1750 proteins were proposed as substrates of HDAC, including p53, GATA1–3, signal transducer and activator of transcription (STAT) 3, STAT5, FOXP3, estrogen and estrogen receptor, HSP90, and α‐tubulin 22. Acetylation of these non‐histone proteins has pleiotropic effects, such as increased expression of FOXP3 22, decreased expression of DNMT1, interference between proteins, and increased p53 transcriptional activity 23. Our results showed that the decreased acetylation rates of H3 and H3K9 might be affected by increased HDAC1 and HDAC5, which inhibits the transcriptional activity of regulatory T cells and reduces the number of Tregs. However, the contrary result has been reported that H3K9 was increased in AAA patients 13.

## Conclusions

The rates of DNA methylation and histone modifications of Tregs in peripheral blood were altered in AAA patients compared with healthy subjects. However, there is still controversy regarding the increase or decrease of H3K9 in AAA patients. Thus, further studies are needed to settle this discrepancy.

## Conflict of interest

The authors declare no conflict of interest.

## Author contributions

QX contributed significantly to analysis and manuscript preparation. JZ contributed to the conception of the study. YH performed the data collection and collation. XZ performed the data analyses. HJ, YL, XW, QG, ZL, DB and ZD participated in some experiments. SX wrote the manuscript.
